# Behavior of a Precast Bridge Pier with Basalt Fiber-Reinforced Polymer (BFRP)-Strengthened Segments under Seismic Loading

**DOI:** 10.3390/polym16142018

**Published:** 2024-07-15

**Authors:** Chao Li, Yaowei Peng, Pengyu Yang, Hao Zhou, Ningbo Wang

**Affiliations:** School of Civil Engineering, Central South University, Changsha 410075, China

**Keywords:** basalt fiber reinforced polymer, strengthened, earthquake excitations, precast column

## Abstract

The precast segmental column (PSC) has been proposed for reducing onsite construction time and minimizing impacts on traffic and the environment. It has been proven to have good seismic performance according to previous studies. However, due to the rocking behavior of the column, the toe of the bottom segment could experience excessive compressive damage. In addition, the commonly used steel rebars in the PSC could experience corrosion problems during the service life of the structure. Moreover, ordinary Portland cement concrete (OPC) is normally used in the construction of the PSC, but the manufacturing processes of the OPC could emit a lot of carbon dioxide. This paper investigates the seismic performance of PSCs incorporating Basalt Fiber Reinforced Polymer (BFRP) bars and geopolymer concrete (GPC) segments. To mitigate the concrete crushing damage of the segment, the BFRP sheet was used to wrap the bottom segment of one of the specimens. The results revealed that the BFRP-reinforced geopolymer concrete PSC exhibited good seismic performance with minimal damage and small residual displacement. Strengthening the bottom segment with BFRP wrapping proved to be effective in reducing concrete damage. As a result, the column with BFRP wrap demonstrated the ability to withstand ground motions with higher Peak Ground Acceleration (PGA) compared to the column without strengthening.

## 1. Introduction

Recently, green construction technologies have attracted a lot research interest around the world [1]. Green construction technologies normally consist of a range of sustainable innovations aimed at reducing environmental impact and enhancing building efficiency, which include but are not limited to innovative construction methods and sustainable construction materials. The construction industry worldwide has relied on conventional cast-in-place methods for steel-reinforced concrete structures for many years. However, this approach has demonstrated various drawbacks, including time-consuming processes such as formwork preparation, on-site concrete casting, and concrete curing, leading to traffic disruption. Additionally, unavoidable environmental impacts like dust, water, and noise pollution further compound the issues. To address these challenges, a precast segmental column (PSC) has been proposed and employed as a substructure for bridges to accelerate the construction of bridges [2,3]. In PSC designs, segments are typically fabricated in a factory and assembled on-site using prestressed tendons. The onsite construction time can be reduced significantly. Despite their benefits, the applications of PSCs are limited due to concerns about their seismic performance. However, recent advancements in construction materials and techniques are driving efforts to overcome these limitations and expand the application of PSCs to regions with high seismicity.

In recent years, a series of investigations have been conducted to explore the seismic behavior of PSCs, aimed at broadening their range of applications [4,5,6]. These studies have revealed that PSCs exhibit a superior self-centering capacity compared to cast-in-place columns. For example, Ou et al. [7] revealed that the proposed PSCs possessed excellent drift capacities that could be used in regions of high seismicity. Nevertheless, the presence of openings at the joints may lead to concrete crushing damage near these regions [8]. To address such concerns and mitigate potential damage, various methods have been proposed. For instance, some researchers have adopted high-performance fiber-reinforced concrete to prepare the precast segments [9]. Some researchers have suggested the use of steel [10] or fiber-reinforced polymer (FRP) tubes [11,12] to confine the concrete to the critical areas. Li et al. [8] proposed a seismic resilient PSC with a damage-controllable and repairable plastic hinge, and the test results showed that the concept was feasible. The damage of the proposed column could be repaired easily and the column after retrofit also showed good seismic performance. Tazarv et al. [13] proposed a new hybrid rocking column with external tendons and it was concluded that the design improved the accessibility to repair the column. Shen et al. [14] conducted shake table tests of post-tensioned precast piers with the bases enhanced by a steel jacket and UHPC.

Conventional PSCs are typically constructed using Ordinary Portland cement (OPC) and steel rebars. However, the manufacturing process of cement is well known for its significant carbon dioxide emissions, accounting for more than 5% of global CO_2_ emissions [15]. To address the environmental concerns, the concept of environmentally friendly concrete binders has been introduced. One such alternative is Geopolymer concrete (GPC), a cementless binder comprising aluminosilicate material and alkaline liquids [16]. Commonly utilized aluminosilicate materials in GPC include fly ash and slag, both of which are industrial byproducts, resulting in reduced carbon dioxide emissions and overall cost when compared to conventional cement-based OPC. Considering the economic and environmental advantages, Geopolymer concrete presents a promising alternative to cement-based concrete. Numerous studies have been conducted to explore the mechanical properties of GPC material [17], as well as its application in structural elements such as beams and columns [18]. For the GPC structures under seismic loading, Saranya et al. [19] investigated the behavior of beam–column joints made from GPC under reverse cyclic seismic loading, and they developed finite element models based on the test results. They demonstrated that the geopolymer concrete, when properly reinforced with steel fibers, could provide comparable performance to traditional cement concrete under seismic loadings at potentially reduced cost/strength ratios. Alashker et al. [20] investigated the seismic efficiency of innovative fiber-reinforced polymer (FRP)-recycled aggregate geopolymer concrete (RAGC) steel-tubed columns (FGSTCs). It was concluded that FGSTCs, incorporating FRP, recycled aggregate geopolymer concrete, and specific tube configurations were capable of achieving robust seismic performance. These findings suggest that optimizing the compression ratio and incorporating FRP and recycled materials can enhance energy dissipation capabilities and reduce failure rates compared to traditional concrete structures. Maniarasan et al. [21] investigated the application of Binary Blended Geopolymer Concrete (BBGC) in beam–column joints, emphasizing its potential advantages over conventional cement concrete in terms of sustainability and structural performance under seismic conditions. Raza et al. [22] developed FEA and analytical models to predict the behavior of the GPC members. In summary, GPC could be an excellent alternative to traditional cement-based concrete. Numerous researchers have extensively investigated its material properties, highlighting its potential for reducing the carbon footprint of construction activities. The applications of GPC in various structural members have been explored, demonstrating its suitability for use in beams, columns, and other critical components of buildings and infrastructure. Moreover, the performance of GPC in structures subjected to seismic loading has been a significant area of study. Researchers have found that GPC exhibits promising behavior under earthquake conditions. The combined benefits of environmental sustainability and seismic performance make GPC a compelling choice for future construction projects.

Steel rebars are prone to corrosion, which can lead to the deterioration of steel-reinforced structures. There has been growing interest in using Fiber Reinforced Polymer (FRP) rebars to replace steel bars in the construction of concrete structures [23]. FRP rebars offer several advantages over steel rebars, including higher strength and superior corrosion resistance. Recently, basalt FRP (BFRP) has emerged as an attractive alternative to other FRPs due to its cost-effectiveness, excellent strength, and superior thermal resistance [24]. These advantages have contributed to the increasing popularity of BFRP in various applications [25]. For instance, Issa et al. [24] conducted experimental tests on the shear behavior of beams with BFRP stirrups. Fan and Zhang [26] investigated the performance of BFRP-reinforced inorganic polymer concrete under eccentric compressive loading.

The primary aim of this research was to design precast segmental columns (PSCs) that were environmentally friendly, corrosion-resistant, and seismically resilient by utilizing geopolymer concrete (GPC), basalt fiber-reinforced polymer (BFRP) bars, and BFRP jackets. GPC is a more environmentally friendly alternative to traditional Portland cement concrete. It significantly reduces carbon dioxide emissions, which are a major concern in the cement industry. Many researchers have highlighted the benefits of GPC in terms of sustainability. However, studies of GPC in precast structures under seismic loadings are limited. BFRP bars are non-corrosive, making them ideal for structures exposed to aggressive environments where steel reinforcement would deteriorate. BFRP jackets are effective for the rapid repair and strengthening of existing structures. They enhance the load-carrying capacity and ductility of structural members. Since the damage of the PSCs is always concentrated at the toes of the bottom of precast segments, caused by the rocking of the column, it was decided to use BFRP wrap to strengthen the bottom segment of the PSCs in this study. By leveraging these materials, the current research aimed to explore innovative solutions for sustainable and resilient construction practices of PSCs subjected to seismic loadings. Previous research on PSCs has predominantly focused on their quasi-static performance. However, limited attention has been given to their dynamic performances. Recently, some researchers have conducted shake table tests to investigate the seismic behavior of PSCs [27,28]. Nevertheless, these tests only considered uniaxial excitation, whereas, in reality, earthquake ground motion consists of three components. To fill this research gap, the present study performed shake table tests to assess the seismic performance of BFRP-reinforced GPC segmental columns. In an effort to mitigate concrete damage, a BFRP jacket was applied to strengthen the bottom segment of one specimen, and its dynamic performances were thoroughly examined. This paper presents results of the seismic behavior of PSCs that employ environmentally friendly and corrosion-resistant materials such as GPC, BFRP bars, and BFRP jackets. The findings from this study could contribute to a better understanding of the potential benefits and applications of these emerging eco-friendly and sustainable materials in seismic-resistant precast structures.

## 2. Specimen Designs and Constructions

### 2.1. Design of Specimens

The prototype column [29] was scaled by a length factor of 12 to accommodate the capacity of the shake table system. The scaled-down column had a height and diameter of 600 mm and 100 mm, respectively. The scale factors and scale rules of other physical items are shown in Table 1. Two specimens were constructed and subjected to testing. Both columns shared identical dimensions, with variations arising from the materials used in each specimen. For the first column, named PSC1, GPC and BFRP rebars were employed. Column PSC2 closely resembled PSC1, except for the addition of BFRP fabrics wrapping around the bottom segment to mitigate the concrete damage.

**Figure 1:**
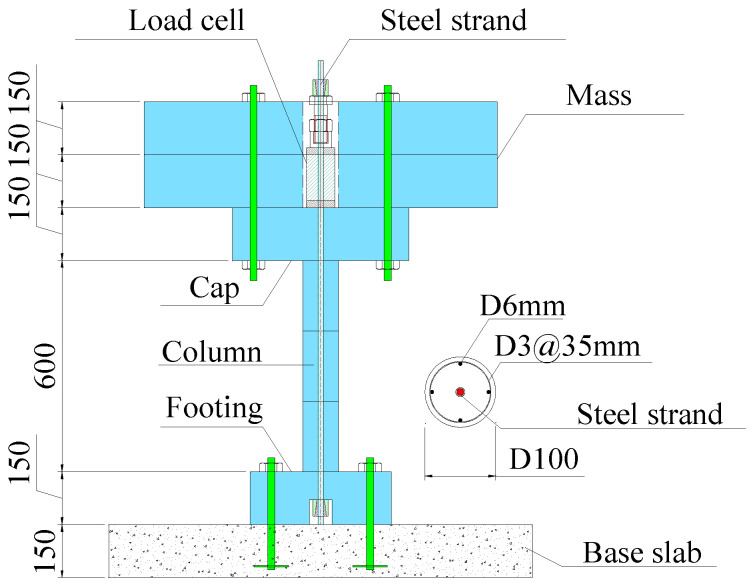
Designs of the specimen.

Figure 1 displays the specimen design details. The specimen consisted of a base slab, the column, and the added mass on top of the specimen. The base slab served as a support for the specimen and also as a platform that integrated four shake tables due to the insufficient carrying capacity of a single table. The top mass was added to mimic the dead load of the superstructure of the bridge system. As illustrated in Figure 1, the top mass, cap, footing, and base slab had the following dimensions: the top mass was 1000 mm in length, 1000 mm in width, and 300 mm in height; the cap measured 500 mm in length, 500 mm in width, and 150 mm in height; the footing had dimensions of 400 mm in length, 400 mm in width, and 150 mm in height, while the base slab had measurements of 1200 mm in length, 1200 mm in width, and 150 mm in height. Each column was divided into three identical segments, each with a height of 200 mm.

For the reinforcement of the segments, four longitudinal bars with a 6 mm diameter were used, and the stirrups had a diameter of 3 mm with a spacing of 35 mm. The resulting longitudinal and transverse reinforcement ratios were 1.5% and 1.1%, respectively. To connect the segments, footing, and cap, post-tensioned steel tendons were employed. The nominal diameter of the tendons was 9.3 mm and the net cross-sectional area of the tendons was 54.7 mm^2^. Both columns had the same design of reinforcement and tendon. As observed in previous studies, the bottom segment of the PSC was prone to experience serious crushing and spalling damages, therefore, for column PSC2, the bottom segment was strengthened by wrapping two layers of BFRP sheet to mitigate the concrete damages during the earthquake. The BFRP had the strength, the thickness, and the elastic modulus of 2100 MPa, 0.12 mm, and 105 GPa, respectively.

### 2.2. Preparation of Specimens

**Figure 2:**
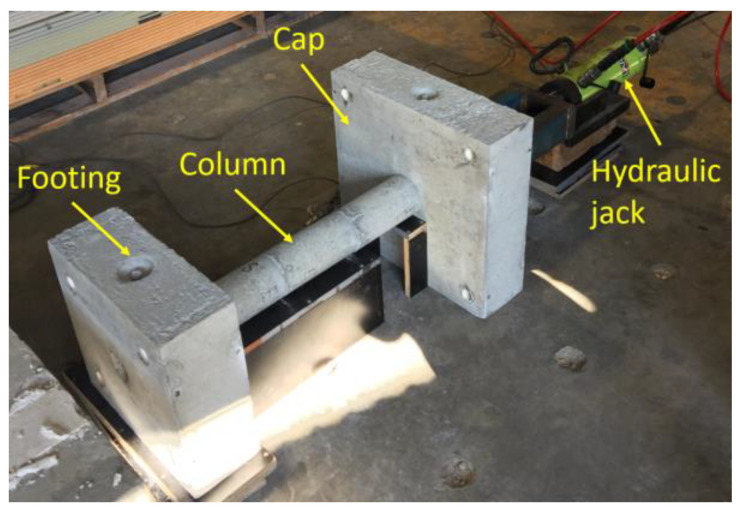
Post-tensioning setup.

The PSC specimens were fabricated in a laboratory by casting the footing, column, and cap separately. Subsequent to the concrete curing process, these components were assembled by clamping the column segments, footing, and cap together using post-tensioned tendons. Figure 2 illustrates the post-tensioning setup. The applied initial post-tensioned (PT) forces in the tendons of PSC1 and PSC2 were 28.3 kN 26.9 kN, respectively.

The mixed design of GPC used in the experiments is presented in Table 2. The actual measured compressive strength of the GPC was 48.5 MPa on the testing day (68 days after casting). It should be mentioned that the columns were cured in air. For the post-tensioned tendon, a steel tendon was employed and its ultimate strength was 1860 MPa. The material properties of the BFRP rebars are summarized and listed in Table 3.

## 3. Test Setup

### 3.1. Installation of the Columns

**Figure 3:**
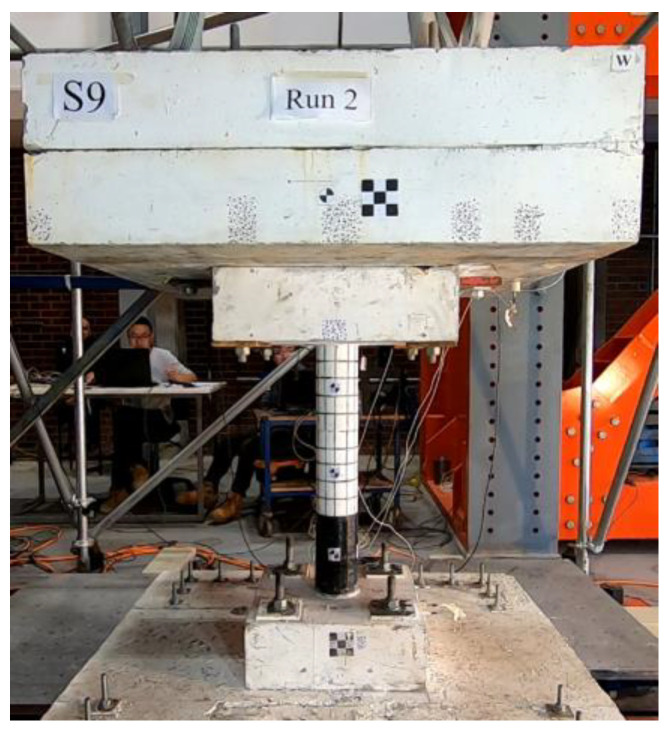
Installed specimen.

To accommodate the shake table’s capacity, four individual shake tables were combined and controlled simultaneously to create a larger unified table. Once the shake tables were ready, the base concrete slab was fixed onto them, and the entire specimen was carefully lifted and installed onto the base slab. The footing of the specimen was securely fastened to the base concrete slab using four bolts. Subsequently, the top mass was properly aligned and fixed to the cap of the column. In consideration of safety, a protection frame was constructed around the top mass, and loose cables were used to connect the mass to the frame. These measures were put in place to prevent any potential hazards in the event of specimen collapse during the test. Figure 3 provides a photo of the final setup of the test, showing the completed arrangement of the specimen and the shake tables.

### 3.2. Arrangement of Sensors

**Figure 4:**
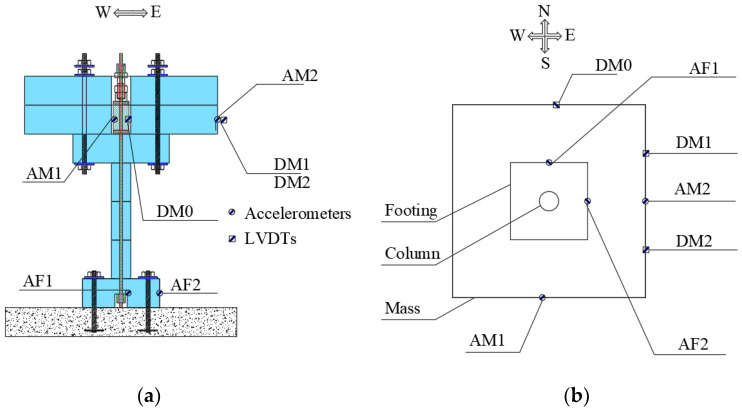
Sensors: (**a**) view from the front, (**b**) view from the top.

During the shake table tests, the dynamic responses of the specimens were monitored using various sensors. Table 4 provides a summary of these sensors. Figure 4 illustrates the placement of the sensors. As shown, two accelerometers (AM1 and AM2) and another two accelerometers (AF1 and AF2) were installed on the mass and footing to capture accelerations of the mass and footing, respectively. For displacement measurements, LVDTs were employed. DL0 was positioned in the North–South (N–S) direction of the mass block, while DL1 and DL2 were placed in the East–West (E–W) direction. In addition to these sensors, the shake table itself featured integrated accelerometers and displacement sensors to cross-check the output accelerations and displacements with the input data. The load cell on top of the cap of the specimen was responsible for monitoring the PT force histories of the tendon during the test. All the sensors were connected to a data acquisition system, and data were sampled at a frequency of 200 Hz. This setup allowed for comprehensive and accurate data collection during the dynamic testing process.

### 3.3. Input Motions

**Figure 5:**
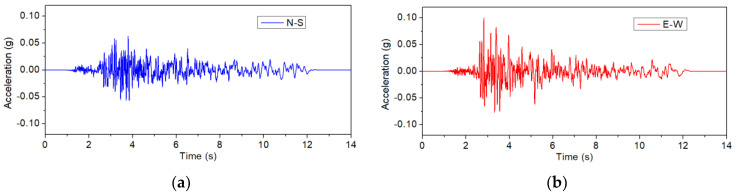
The input motions: (**a**) N–S, (**b**) E–W.

During the test, the specimens underwent a series of bidirectional excitations. The original data obtained from the Niland Fire Station during the 1979 Imperial Valley Earthquake were selected and scaled. For the original data, the peak ground accelerations (PGAs) recorded in the two orthogonal directions were 0.068 g and 0.108 g, respectively. During the tests, the original recorded excitations were scaled and the maximum PGA (0.108 g) was scaled to 0.1 g. The same scale factor was used to scale the input of the orthogonal direction. Subsequently, the maximum PGA was incrementally raised from 0.1 g up to the point of column failure, with a step size of 0.1 g in each interval. Following the similitude law described in Table 1, the time duration of the excitations was compressed by a factor of 3.46. Figure 5 exhibits the input motions in two directions, indicating a maximum PGA of 0.1 g in the E–W direction. In order to obtain the dynamic characteristics of the column during the tests, each formal test was preceded by the application of bi-axial white noise excitations, which had an amplitude of 0.02 g and a duration of 40 s.

## 4. Test Results

### 4.1. Observed Damage Patterns

**Figure 6:**
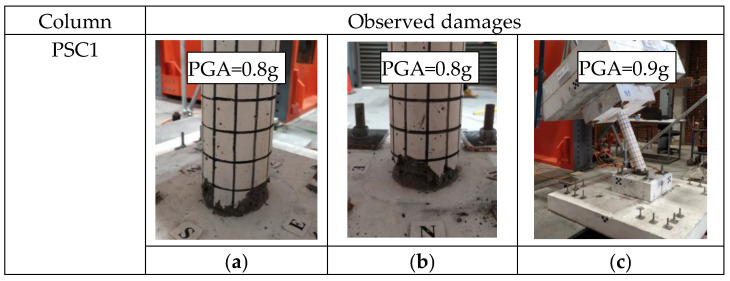

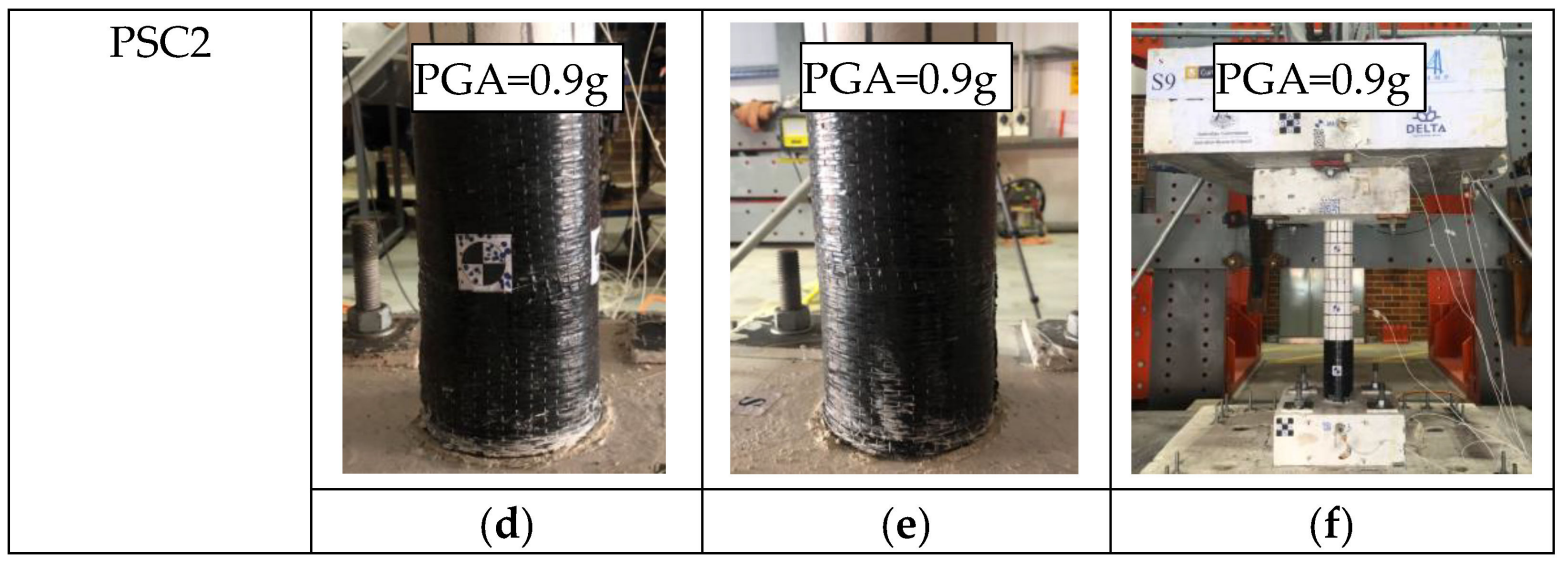
Damage patterns of the specimens: (**a**–**c**) damage of the PSC1; (**d**–**f**) damage of the PSC2.

During the tests, column PSC1, which used BFRP bars-reinforced GPC, collapsed when subjected to a PGA of 0.9 g. On the other hand, column PSC2, with the bottom segment strengthened by BFRP wrap, did not fail even when the PGA reached 0.9 g. Figure 6 depicts the damage experienced by the specimens at the PGAs just before collapse and after collapse. It should be noted that PSC2 was not tested up to collapse.

For column PSC1, it can be observed that the damage was predominantly concentrated at the joint between the column and the footing. This concentration occurred due to the rocking motion of the column, resulting in a reduced contact area between the segment and the foundation. Consequently, the concrete near the toes of the bottom segment experienced significant compressive stress, leading to concrete crushing and spalling damages. The column finally collapsed due to insufficient resistant to the moment from the top mass that was induced by the excitations. The column PSC2 strengthened with BFRP wrap did not collapse until the end of the test when the PGA reached 0.9 g. Column PSC2 was designed to minimize the concrete damages of the segment by strengthening the bottom column with BFRP fabrics. As shown in Figure 6d,e, column PSC2 with BFRP strengthening did not experience severe damages and it could undergo higher excitations compared to column PSC1, indicating that the use of BFRP to confine the segment could effectively mitigate the concrete compressive damage of the concrete and thus improve the seismic resistance of the precast segmental column. In summary, from the damage patterns of the two columns, it was found that the column with BFRP reinforced GPC had localized concrete damages due to the rocking of the column, which was similar to those columns with steel-reinforced OPC [9]. Such damage finally resulted in the collapse of the column at a PGA of 0.9 g. In contrast, for column PSC2, the damage of the segment was minimized by the use of BFRP wrap, which also helped the column to withstand higher PGA without collapse. Therefore, it is effective to use BFRP wrap to strengthen the PSC and improve its seismic performance.

### 4.2. Change of Vibration Periods

**Figure 7:**
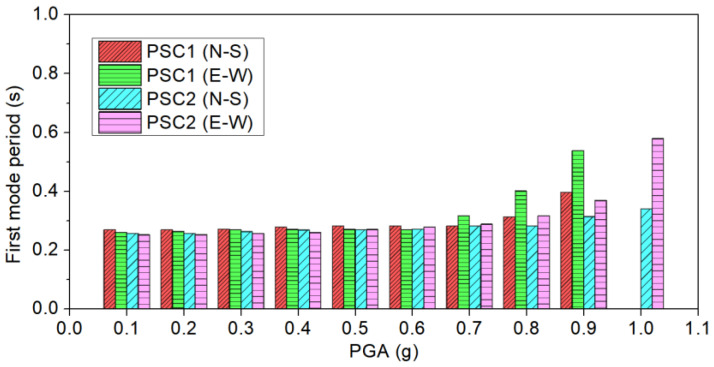
Fundamental periods of PSC1 and PSC2 (before each formal test).

As mentioned before, prior to each formal test, a white noise test was conducted to obtain the vibration period of the column. The changes in the first vibration periods of the specimens are illustrated in Figure 7. It was evident that, until the peak ground acceleration (PGA) reached 0.6 g, there was only a minor increase observed for both columns. This implies that the damage to the two columns was insignificant, indicating that the columns behaved in an almost linearly elastic manner under these PGAs. After reaching 0.6 g, the vibration period of column PSC1 started to increase noticeably. Figure 7 also revealed that, for PSC1, the vibration periods in both horizontal directions were nearly the same when subjected to a relatively low PGA, which is a result of the column’s symmetric design. However, when damage occurred, the vibration period in the E–W direction became longer than in the N–S direction. This is attributed to the fact that the PGA in the E–W direction was higher than that in the N–S direction. Consequently, more severe concrete damage occurred in the E–W direction, leading to reduced lateral stiffness and a longer vibration period. The same observation was found for column PSC2. For column PSC2, the increase in vibration period was slower than that of column PSC1. As shown, after 0.7 g, the vibration period of column PSC2 started to increase gradually, which indicated that the column PSC2 began to experience damage. However, it was found that the increment was less than that of column PSC1. The results clearly demonstrate that the use of BFRP fabrics effectively mitigated damage to the segments.

### 4.3. Displacement Responses

**Figure 8:**
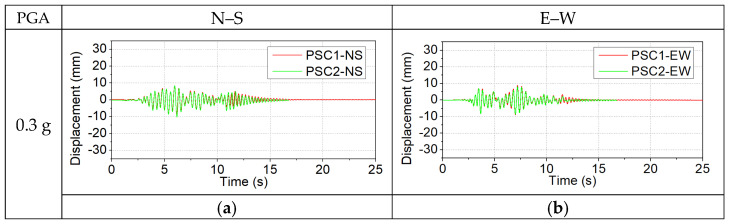

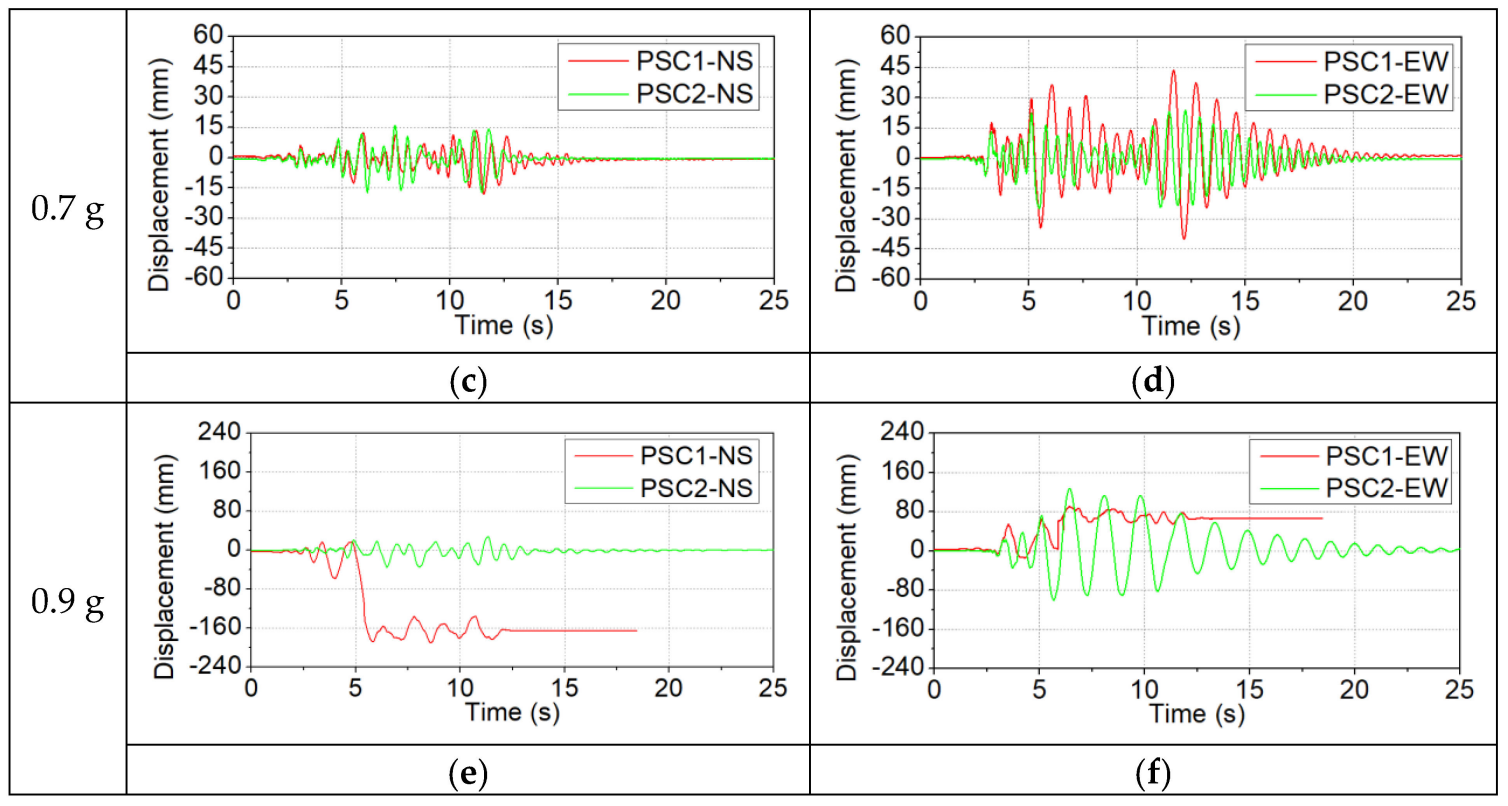
Displacement responses of the columns: (**a**,**b**) 0.3 g; (**c**,**d**) 0.7 g; (**e**,**f**) 0.9 g.

The displacement responses of the specimens were acquired through measurements obtained from the LVDTs. The computation of relative displacement responses was achieved by subtracting the input displacements of the shake table from the measured displacement responses. The responses of the columns under representative small (0.3 g), medium (0.7 g), and large (0.9 g) PGAs were compared here. Figure 8 presents the relative displacements of the two columns.

As shown in Figure 8a,b, under a PGA of 0.3 g, the maximum displacement response of the two columns was approximately 10 mm, which was a relatively small value. In addition, it was also found that the displacement response histories of the two columns were close to each other. This could be attributed to the fact that at small PGAs, the two columns were in their elastic stages, and the concrete did not experience significant plastic deformations. Thus, the BFRP wrap had limited improvement on the performance of column PSC2. When the PGA reached 0.7 g, the displacement responses of the test specimens were shown in Figure 8c,d. Specifically, in the N–S direction, both columns exhibited relatively small displacement responses. The maximum displacement was approximately 15 mm. In contrast, obvious discrepancies were found between the displacement responses of columns PSC1 and PSC2 in the E–W direction. Column PSC1 had much larger displacement responses as compared to column PSC2. The peak displacement of PSC1 reached about 43.7 mm, while the peak displacement of PSC2 was −25 mm. This indicates that the column PSC1 experienced obvious damage, while relatively less damage was experienced by column PSC2, therefore, the displacement response of PSC2 was smaller than that of column PSC1. When the PGA reached 0.9 g, as shown in Figure 8e,f, column PSC1 collapsed due to excessive concrete damage in the segment, while column PSC2 survived under this PGA. As the bottom segment of PSC2 was strengthened by the BFRP jacket, this result demonstrated that the use of the BFRP jacket to strengthen the segment could effectively minimize the concrete damage, thus reducing the displacement responses of the precast segmental column and delaying the collapse of the whole structure.

**Figure 9:**
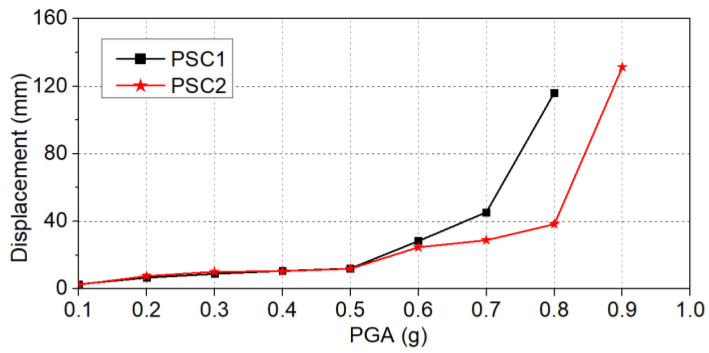
Resultant displacement responses of the columns.

Figure 9 depicts the maximum resultant displacement responses exhibited by the specimens when subjected to various magnitudes of PGAs. Notably, under low-level PGAs, the resultant displacement responses of the two specimens were similar. This observation aligns with previous explanations, wherein both columns remained undamaged when subjected to low-level PGAs. Therefore, the effect of the BFRP wrap was not significant, and both columns performed similarly. When the PGAs were larger than 0.5 g, column PSC1 experienced larger resultant displacement. For example, at a PGA of 0.6 g, the resultant displacement of column PSC1 was 28.4 mm, while the value of column PSC2 was 24.6, which was 13.4% smaller than that of column PSC1. At 0.8 g, such a difference became even more significant and the resultant displacements of columns PSC1 and PSC2 were 116.2 mm and 38.3 mm, respectively. Again, this is attributed to the BFRP jacket-strengthening of the bottom segment of PSC2, which significantly reduced the damage and thus reduced the displacement responses. At 0.9 g, column PSC1 collapsed due to excessive damage to the bottom segment, and column PSC2 also experienced a large resultant displacement response. The maximum resultant displacement of PSC2 reached 131.4 mm. This significant increase could be attributed to the localized failure of the BFRP jacket and damage to the inside concrete. In summary, the use of the BFRP jacket to strengthen the bottom segment reduced the damage to the concrete, therefore, the column PSC2 could undergo higher PGAs without collapsing.

### 4.4. Histories of PT Forces

**Figure 10:**
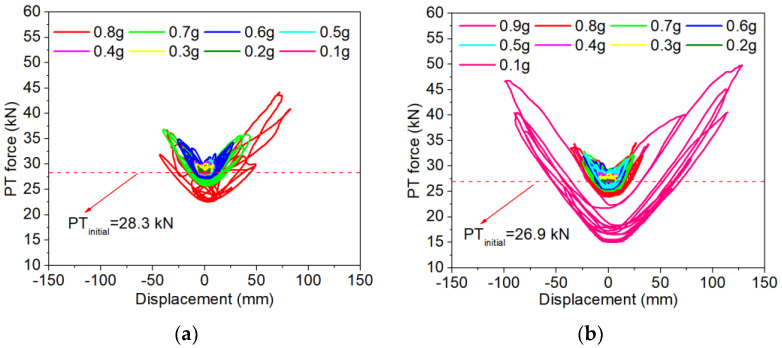
PT forces in the tendons of the columns: (**a**) PSC1; (**b**) PSC2.

The PT force histories were recorded by the load cell installed underneath the anchor of the tendon during the experiments. Figure 10 illustrates the PT force histories in relation to the lateral displacements in the E–W direction of the columns. Table 5 summarized the maximum and minimum PT force of the two specimens. It is evident that a general V-shaped relationship is observed between the PT forces and the lateral displacements. This behavior can be attributed to the positioning of the PT tendon at the column’s center. The deformation of the column resulted in joint openings at the joints, which then caused the elongation of the tendon. When the column returned to the zero-displacement position, the PT force also decreased and reached its initial applied value. It should be noted that with the increase in the PGAs, the column experienced concrete damage, which could result in PT force loss after the test. As shown in Figure 10a, column PSC1 exhibited a peak post-tension force of 44.2 kN at the lateral displacement of 72.8 mm when subjected to a PGA of 0.8 g. For column PSC2, the use of BFRP wrap to strengthen the bottom segment enabled the column to withstand higher PGAs. The column also had larger maximum lateral displacements, which in turn led to a larger PT force during the test. As illustrated, the maximum PT force reached 49.8 kN when subjected to a PGA of 0.9 g, with a corresponding lateral displacement of 129.1 mm.

It should be noted that both columns experienced PT force loss after the test. For column PSC1, the residual PT force in the tendon after 0.8 g was 22.6 kN, and the PT force loss was about 20.19%, as referred to in the initial applied PT force. In comparison, the residual PT force of PSC2 at 0.8 g was 24.0 kN, and the corresponding PT force loss was 10.72%. The PT force loss of PSC2 was less than that of column PSC1. As discussed above, the BFRP wrap effectively minimized the concrete damage of the column, which therefore reduced the PT force loss.

### 4.5. Discussion

From the test results, it can be found that the PSCs showed good seismic performance. Similar to the shake table tests that were conducted in previous studies [28], the PSCs experienced small residual displacement as compared to the traditional monolithic columns. In addition, the damage of the BFRP-reinforced GPC column performed well throughout the test, showing a similar damage pattern to that of the OPC precast columns. This shows that the GPC and BFRP bars could be alternatives to the OPC and steel bars for the construction of PSCs, respectively. The damage to the PSCs was the concrete compressive damage that concentrated at the toes of the precast segment, which was mainly due to the rocking of the column. While the damage to the monolithic column was the compressive and tensile damage of the concrete and also the yielding of the longitudinal steel reinforcement. As demonstrated in the test results, the use of BFRP wrap effectively mitigated the compressive damage to the segment. Previous studies have shown that CFRP jacketing was effective in reducing the damage to the segmental bridge columns [30]. Some studies also adopted a steel jacket to minimize the concrete damage of the PSCs [10]. However, the CFRP is more costly as compared to BFRP, and the steel jacket had the issue of corrosion. In this study, it was proven that the more cost effective BFRP could also reduce the damage of the PSCs subjected to dynamic seismic excitations. BFRP is known for its high strength and durability. Its ability to resist corrosion can indeed contribute to reducing long-term maintenance and replacement costs while enhancing the seismic resilience of the PSCs.

## 5. Conclusions

This research conducted a series of shake table experiments to explore the seismic behavior of two PSCs. The primary objectives were to investigate the potential application of BFRP bars and GPC in PSCs, and evaluate the efficacy of strengthening the precast segment with BFRP wrap to mitigate the damage to the column. Based on the test results, some conclusions were drawn as follows:

(1) The experimental findings demonstrated that precast columns featuring BFRP-reinforced GPC segments exhibited good seismic performance when subjected to biaxial seismic excitations. The damage was limited to the bottom segment only.

(2) Both columns had small residual displacement during the tests due to the use of unbonded PT tendons. Such characteristics could make post-earthquake retrofitting activities easier since it could be difficult or even impossible to repair the columns if the residual displacement is too large.

(3) The use of BFRP to strengthen the bottom segment of PSC2 effectively reduced the damage to the concrete.

(4) Due to reduced damage in column PSC2, slower and smaller increments of the vibration period were observed.

(5) As the column PSC2 had less damage, it had smaller displacement responses at large PGAs. Additionally, column PSC2 also underwent earthquake excitations with higher PGAs as compared to the reference column PSC1, and did not collapse even at the PGA of 0.9 g.

(6) Both columns experienced PT force increases when the columns deformed laterally. PT force losses were also found in both columns. Due to the use of the BFRP strengthening technique in PSC2, there was much smaller PT force loss when the column was subjected to the PGA of 0.8 g.

## Figures and Tables

**Table 1:** Scale rules and factors.

Physical Items		Scale Rules	Scale Factors
Geometry	Length (*l*)	*S_l_*	12
	Displacement (*δ*)	*S_l_*	12
Material properties	Elastic modulus (*E*)	*S_E_*	1
	Stress (*σ*)	*S_E_*	1
	Strain (*ε*)	1	1
	Poisson’s Ratio (*ν*)	1	1
Dynamic properties	Acceleration (*a*)	*S_a_*	1
	Mass (*m*)	*S_E_S_l_* ^2^ */S_a_*	144
	Frequency (*ω*)	*(S_a_/S_l_)* ^0.5^	0.29
	Velocity (*v*)	*(S_l_S_a_)* ^0.5^	3.46
	Time (*t*)	*(S_l_/S_a_)* ^0.5^	3.46
Loadings	Force (*F*)	*S_E_S_l_* ^2^	144
	Moment (*M*)	*S_E_S_l_* ^3^	1728

**Table 2:** Mixed design of concrete (unit: kg/m^3^).

Mixed Concrete	Aggregates	Sand	Fly Ash	Slag	Na_2_SiO_3_ Solution	NaOH Solution
GPC	1196	644	360	40	173.7	59.4

**Table 3:** Material properties.

Material	Diameter	Elastic Modulus	Yield Strength	Ultimate Strength
	(mm)	(GPa)	(MPa)	(MPa) Steel/BFRP
Longitudinal rebar	6	55	-	1100
Stirrup	3	55	-	1100
Tendon	9.3	195	1674	1860
BFRP fabric	-	105	-	2100

**Table 4:** Sensor details.

Device	Name	Note
Accelerometers	AM1	Acceleration at mass (N–S)
	AM2	Acceleration at mass (E–W)
	AF1	Acceleration at footing (N–S)
	AF2	Acceleration at footing (E–W)
LVDTs	DM0	Displacement at mass (N–S)
	DM1	Displacement at mass (E–W)
	DM2	Displacement at mass (E–W)
Load Cell	FT	Force in the tendon

**Table 5:** Summary of the PT forces.

PGA (g)	PSC1			PSC2		
	Max (kN)	Min (kN)	Loss (%)	Max (kN)	Min (kN)	Loss (%)
0	28.28	28.28	0.00	26.90	26.90	0.00
0.1	28.37	28.27	0.07	27.03	26.90	0.01
0.2	29.53	28.16	0.44	27.35	26.88	0.05
0.3	30.08	28.04	0.88	28.44	26.84	0.21
0.4	30.66	27.95	1.17	28.65	26.72	0.67
0.5	30.91	27.86	1.51	29.96	26.58	1.18
0.6	34.92	27.20	3.83	31.86	25.93	3.60
0.7	36.82	25.75	8.94	32.88	25.02	7.00
0.8	44.18	22.57	20.19	34.53	24.01	10.72
0.9	-	-	-	49.83	15.00	44.22

## Data Availability

Data will be made available on request.
